# Is She or He the Key Player in Pickleball Mixed Doubles? A Pilot Study on Sex-Based Performance Profiles

**DOI:** 10.3390/sports13110397

**Published:** 2025-11-06

**Authors:** Alexandru Nicolae Ungureanu, Paolo Riccardo Brustio, Damiano Li Volsi, Corrado Lupo

**Affiliations:** 1 Department of Life Sciences and Systems Biology, University of Turin, 10126 Turin, Italy; alexandru.ungureanu@unito.it; 2 Neuromuscular Function Research Group, School of Exercise and Sport Science, University of Turin, 10126 Turin, Italy; damiano.livolsi@unito.it (D.L.V.); corrado.lupo@unito.it (C.L.); 3 Department of Clinical and Biological Sciences, University of Turin, 10126 Turin, Italy; 4 Department of Medical Sciences, University of Turin, 10126 Turin, Italy

**Keywords:** pickleball, racket-sports, performance analysis, sex differences

## Abstract

Pickleball is a recent sport, and very little scientific information exists on its match performance, especially for mixed doubles. Therefore, the aim of this study was to describe the performance profile in relation to sex differences in terms of shot outcome, margin of victory, and advantage. Seventeen elite mixed double matches from Professional Pickleball Association tours were analyzed, specifically considering the final as well as the penultimate shot with regard to the sex of the opponent who played it to analyze the inter-player dynamics between the opponents. Elite mixed pickleball matches have been characterized by 1:1.4 work-to-rest ratio, with rallies meanly lasting 10.6 s and separated by 11.4 s between them. Among the 1678 final shots analyzed, males played significantly fewer forced errors and more winners than females, especially when the penultimate shot was played by a female opponent. No sex differences emerged when the penultimate shot was played by a male opponent. Additionally, males played more winners both when leading and when winning the match. Mixed doubles pickleball matches appear to be partially influenced by sex-imbalanced game dynamics, with a higher number of winners played by males, particularly when the preceding shot is played by a female, suggesting that this format offers only moderate inclusivity between sexes.

## 1. Introduction

Pickleball is a racket sport that has experienced rapid growth in recent years, particularly in North America [1,2]. Although invented in 1965, it became formally organized in 1984 with the establishment of the United States Pickleball Association (USAPA). Since the 2000s, USAPA and other international associations such as the International Pickleball Federation (IPF) and the European Pickleball Federation (EPF) have promoted the sport’s development nationally and worldwide [3,4]. Alongside national and international growth in participation, competitive pickleball began to take off, and by 2020 the Professional Pickleball Association (PPA) was formed to organize high-level tournaments and manage player rankings. To date, the PPA Tour 2024 includes 26 tournaments across 16 states, offering both professional and amateur competition [5].

Similarly, Major League Pickleball, created in 2021, aims to expand participation to 40 million people globally by 2030 [6,7]. These developments have significantly increased prize money for professional players, reaching $5.5 million in 2023 [7].

Technically and tactically, pickleball blends elements of tennis, table tennis, and badminton. It is played in singles or doubles (same-sex or mixed) on a 13.4 × 6.1 m court using a 40 × 20 cm paddle made of graphite, fiberglass, or carbon (i.e., slightly larger than a ping-pong paddle) and a perforated plastic ball (~21 cm circumference). Matches are typically best-of-three sets to 11 points, with players serving until they lose a rally or commit a fault [8]. However, specific regulations apply to pickleball doubles. Notably, in pickleball, compared to tennis, both players on a doubles team serve in sequence until the team loses the rally and a side-out occurs, with the server starting on the right-hand side when their own score is even and on the left-hand side when it is odd. This dynamic serving system introduces tactical variability absent in tennis doubles, where each player serves for an entire game in a fixed rotation regardless of the score. However, teams may choose to have the male player serve first from the right side of the court as a strategic decision, aiming to leverage potentially stronger serves and gain an early advantage in the rally, even though this is not part of the official USA Pickleball rules [8].

To date, few studies have explored technical and tactical performance separately for sex, investigating effectiveness trends by rally type and server-versus-returner role [9], shifts in serve effectiveness [10], and match activity profiles [11]. Time-motion analysis revealed that pickleball matches are shorter than in badminton, tennis, and padel, while rally and rest durations are similar to padel [11]. Furthermore, pickleball likely demands rapid lateral and forward–backward footwork movements characterized by accelerations and decelerations, largely due to its smaller court dimensions compared to other racket sports, such as tennis or padel. This reduces the distance players must cover, especially in doubles play. In addition, in double matches in racket sports, technical and tactical strategies require players to cooperate with each other while also closely monitoring their opponents’ strokes [12].

Technical and tactical performance are especially relevant in mixed doubles, where both cooperation and sex-based dynamics can influence performance outcomes. In badminton, for example, tactics take priority over other indicators like rally durations and stroke counts [13]. In fact, competing man vs. female could represent a significant imbalance from both sides, as male players outperform female players in certain scenarios (e.g., within the first four strokes) and the involvement of male players against female opponents has also been shown to significantly influence the outcomes of mixed doubles table tennis matches [12]. Historically, mixed doubles tennis matches were influenced by etiquette norms (e.g., males were expected to take most of the shots, including the more difficult and winning ones, and control the shots directed at female opponents) based on social rules that reflected a strong patriarchal and paternalist ideology [14]. Tactical interactions were governed by self-imposed restrictions that limited competitiveness, reducing mixed doubles to a recreational level rather than a competitive one [14]. Furthermore, from a strategic perspective, it was suggested that the female players should allow the man to take on the role of aggressor and primary point scorer, limiting her involvement to delivering the serve directed toward her [15]. In recent times, not much has changed. In the modern era, mixed doubles tennis competitions have continued to be regarded as “just for fun” events, even in major tournaments like Wimbledon [15]. However, in pickleball, the most powerful shots, such as the serve and the smash, which may favor male players, are the least frequently used shot types [11].

Despite the increasing relevance of mixed doubles in competitive pickleball, no specific data are currently available for this format. Given its recent emergence, unique dynamics may characterize this division (e.g., final shot winners more often performed by males against female opponents). Moreover, different patterns could emerge when investigating contextual factors, such as margin of victory (MoV) (i.e., winning the match) and margin of advantage (MoA) (i.e., leading in the running score). As in studies in other sports [16,17], these patterns provide complementary insights. The first one captures the overall outcome of the match, while the second reflects situational dominance and tactical control during rallies. Therefore, this study aimed to describe the technical and tactical, and time-motion performance profile of mixed doubles pickleball, in general, and in relation to sex, shot efficacy (including technical and tactical aspects), and contextual factors such as margin of victory and advantage. Specifically, it investigated whether winners, forced errors, and unforced errors differed between the female and male partners.

## 2. Materials and Methods

### 2.1. Design and Instruments

In this study, a notational analysis was performed on 20 elite pickleball pairs within several tournaments of the PPA tours. Specifically, a sample of 17 elite mixed matches from the PPA Tour between 2023 and 2024 was analyzed. Video footage was recruited online from the PPA Tour YouTube channel [18]. The sample included a total of 3356 final and penultimate shots (corresponding to 1678 rallies) and 404 points scored. Since the data were based on publicly available resources, no informed consent was obtained.

Notational analysis was conducted by three analysts using Longomatch Open Source software (version 1.3.2). Since this study was based on an observational notational analysis process using publicly available matches, a formal a priori power-based sample size estimation was not feasible. However, to provide a reliable analysis, either intra- or inter-observer reliability was assessed. In particular, each observer evaluated the same KPIs (i.e., Key Performance Indicator) for two randomly selected sets of play, with a 14-day interval between assessments. The repeated analyses for each observer demonstrated good to excellent grades of agreement (i.e., intra-class, ICC range = 0.91–0.99), as well as between individual observations in both sessions (inter-class, ICC range = 0.89–0.97), for all KPIs [19].

### 2.2. Procedures

Eight technical, tactical, and time-motion KPIs were examined, similarly to previous studies of racket sports [20,21,22] (see Supplementary Table S1). Specifically, from the technical and tactical perspective, final shots (i.e., last shot of each rally) were analyzed in relation to their outcome (i.e., winner, forced error, or unforced error), match context (i.e., margin of victory and margin of advantage), and sex of the executing player. According to previous studies in racket sports [23,24], a winning shot occurs when a player wins a point with a direct and valid stroke (e.g., an effective dink that lands just out of reach, a drive that forces the ball to bounce twice in the opponent’s court, or a shot that strikes the opponent’s body before going out). Conversely, a forced error happens when a player loses the point by missing a difficult shot executed under pressure or from a poor position (e.g., a missed volley while off-balance near the kitchen line or failing to return a deep topspin serve) while an unforced error occurs when a player loses the point through a mistake in an easy situation, with sufficient time and space for proper execution (e.g., a routine dink sent into the net, overhitting an open-court winner, or stepping into the non-volley zone on an unpressured volley). Additionally, the final shot was examined in relation to the penultimate shot, considering the opponent’s sex, to explore inter-player dynamics.

### 2.3. Statistical Analysis

Descriptive statistics was applied for the 8 technical and tactical and TMA variables, and the data are presented as mean ± standard deviation. Results are presented as percentages according to Formula (1).

Formula (1). Calculation of final shot outcome percentages: the number of each type of final shot (Forced Errors, Unforced Errors, Winners) performed by each sex (Male, Female) under each contingency (Overall, Penultimate Female, Penultimate Male), divided by the total number of final shots by the same sex under the same contingency, multiplied by 100.

(1) $$\frac{Number\;of\;final\;shot\;outcome\;\left(FE,UE,W\right)\;\;in\;relation\;to\;sex\;\left(M,F\right)\;under\;each\;contingency\;\left(O,PF,PM\right)\;\;}{Total\;number\;of\;final\;shot\;in\;relation\;to\;sex\;\left(M,F\right)\;under\;the\;same\;contingency\;(O,PF,PM)}\times 100$$

After verifying normality with the Kolmogorov–Smirnov normality test, paired-samples *t*-tests were applied, and significance level was set at *p* ≤ 0.05. Effect sizes were reported as Cohen’s d, considering effect sizes as small (0.2–<0.6), medium (0.6–<1.2), large (1.2–<2.0), very large (2.0–<4.0), or extremely large (≥4.0) [25].

## 3. Results

Descriptive time-motion values, recorded during the 1678 analyzed rallies, have been reported in Table 1.

Table 2 describes the values (%) of male and female final shot outcomes. In particular, forced and unforced error and winner final shots were reported both overall (regardless of the penultimate shot) and separately according to sex, considering (i) all the shots, (ii) only the shots of the pair that won the match, and (iii) only the shots made by the pair leading in the running score.

### 3.1. Overall Scenario

Paired-samples *t*-tests revealed significant sex differences in several shot categories (see Supplementary Table S2). Overall (i.e., regardless of the penultimate shot), males committed significantly fewer forced errors than females (mean difference = −0.11 ± 0.03, t(16) = −3.41, *p* = 0.004, d = −0.83), while unforced-error rates did not differ significantly (t(16) = 0.24, *p* = 0.812, d = 0.06). Conversely, males produced a significant higher proportion of winners (mean difference = 0.10 ± 0.02, t(16) = 4.10, *p* < 0.001, d = 1.00). When the penultimate shot was played by a female opponent (i.e., PF condition), males still played fewer forced errors (mean difference = –0.05 ± 0.02, t(16) = −2.20, *p* = 0.043, d = −0.53), while unforced errors remained equivalent (t(16) = −0.08, *p* = 0.940). Similarly to the overall results, males still finished more winner final shots (mean difference = 0.08 ± 0.02), t(16) = 3.31, *p* = 0.004, d = 0.80). On the other hand, when the penultimate shot was played by a male opponent (i.e., PM condition), the sex gap in forced errors narrowed to a non-significant trend (t(16) = −1.96, *p* = 0.067, d = −0.48), as well as for unforced error (t(16) = 0.78, *p* = 0.448) and winner final shots (t(16) = 1.53, *p* = 0.146).

### 3.2. “Winning the Match” Scenario

Paired *t*-tests limited to the shots hit by the pair that ultimately won the match showed only one reliable sex difference (see Supplementary Table S3). Overall (i.e., independent to the penultimate shot), male players produced significantly more winner final points than female players (mean difference = 0.205 ± 0.042, t(16) = 4.93, *p* < 0.001, d = 1.20). No significant sex differences were observed in forced (t(16) = −1.62, *p* = 0.125) and unforced (t(16) = −0.26, *p* = 0.801) errors. Considering the penultimate shot in PF condition, no significant sex effects emerged for forced errors (t(16) = −1.28, *p* = 0.219), unforced errors (t(16) = −0.38, *p* = 0.713), or winners (t(16) = 1.37, *p* = 0.190). Similarly, when the penultimate shot was set in PM condition, no sex-based differences were found in forced errors (t(16) = −0.48, *p* = 0.641), unforced errors (t(16) = 0.60, *p* = 0.556), or winners (t(16) = 0.94, *p* = 0.362).

### 3.3. ”Running Score Advantage” Scenario

Paired *t*-tests limited to the shots hit by the pair that was ahead in the running score showed a slightly different pattern compared to the results based on match winners (see Supplementary Table S4). Overall (i.e., penultimate-shot independent), males recorded a higher winner rate than females (mean difference =0.078 ± 0.034, t(16) = 2.30, *p* = 0.035, d = 0.56) while forced (t(16) = −1.23, *p* = 0.237) and unforced errors (t(16) = −0.73, *p* = 0.475) did not differ. Considering a penultimate shot by an opposing female (i.e., PF condition) the winner advantage for males persisted (mean difference = 0.061 ± 0.028), t(16) = 2.14, *p* = 0.048, d = 0.52), whereas forced- and unforced-error gaps remained non-significant (both *p* > 0.36). On the other hand, considering a penultimate shot by an opposing male (i.e., PM condition), no sex effect emerged. Specifically, forced errors (t(16) = −0.36, *p* = 0.725), unforced errors (t(16) = −0.06, *p* = 0.953), and winners (t(16) = 1.89, *p* = 0.077) were not significantly different.

## 4. Discussion

To our knowledge, this is the first study to quantify the technical and tactical, and time-motion performance profile in mixed doubles pickleball, with a specific focus on sex-related differences, shot efficacy, and margins of victory and advantage, including the role of the penultimate shot in terms of player sex. The main findings indicate that male players concluded more rallies with winning final shots, particularly when the point had been set up by a female opponent and committed fewer forced errors overall. Nonetheless, error rates were statistically comparable between sexes under favorable conditions, such as when leading in the running score or winning the match. These results suggest that mixed doubles pickleball matches are partially influenced by sex-based imbalances in game dynamics, making them only moderately inclusive and reflecting a subtle continuation of historically gendered patterns of play.

In general, the description of the time-motion profile in mixed doubles showed to be consistent with previous literature in males’ doubles pickleball, both in terms of volume [e.g., total match duration (36 vs. 36 min), effective playing time (17 vs. 14 min), average rally duration (11 vs. 11 s)] and density [i.e., work-to-rest ratio (1:1.4 vs. 1:1.6)] [11]. In comparison to other racket sports such as padel, match durations lasted considerably less time, representing times of approximately half and a third of the length compared to sub-elite and elite padel matches, respectively [20,21], while effective playing time is more similar to sub-elite (i.e., 17 min) than elite padel (i.e., 30 min). In terms of effective playing time, the average rally duration lies between sub-elite (i.e., 7 s) and elite (i.e., 13 s) values. On the other hand, the density analysis reveals a low work-to-rest ratio of just 1:1.4, even lower than what is typically observed in tennis (i.e., 1:3 to 1:5) and padel sub-elite (1:3.4) and elite (i.e., 1:2) [20,21,22]. This effect may be due to the smaller court area (almost three times smaller than a doubles tennis court and twice as small as a padel court) which allows players to retrieve the ball and reposition for the next serve rapidly, making it very easy to restart the play. In addition, the smaller court leads players to cover shorter distances and often finish rallies within just a few shots (e.g., up to eight), mostly executed near the non-volley line, while also requiring faster decision-making and greater precision due to the tighter playing space [9].

From a technical and tactical standpoint, three key findings emerged. First, male players finished more points with winners across all the contexts examined (i.e., overall, when the pair was leading, and when they ultimately won). The percental difference was large overall (approximately a 10% gap) and doubled when considering winning pairs (approximately a 20% gap), while remained moderate when considering those holding a running score advantage (approximately 8% gap). Second, sex differences in error production were context-dependent. Females committed more forced errors overall. However, this trend disappeared when considering only winning pairs and those who were ahead in the running score. Third, the sex of the opponent’s penultimate shot influenced the outcome of the rally. Specifically, when the penultimate shot came from an opposing female (PF condition), males still produced more winners and fewer forced errors, while when it originated from an opposing man (PM condition), sex differences were all non-significant.

The first observed pattern suggests that, at an elite level, mixed-pickleball tactics tend to position male players as the primary point scorer (medium ES). Similar sex differences have already been reported in other racket sports such as table tennis [12], where male players outperform female players, especially within the first strokes, and males facing females had a greater impact on match outcomes. However, this “male finishing bias” is mitigated in table tennis by the rule requiring all four players to alternate shots, which helps ensure a more balanced competition [12]. In contrast, this rule is absent in pickleball, and it may contribute to the observed performance imbalance, potentially favoring the biologically stronger member of the pair. Moreover, despite the potential advantage that powerful shots like the serve and smash may confer to males over females, they remain the least employed shot types [11], likely due to the restricted ball velocity resulting from its perforated plastic design. This pattern also supports ongoing shifts in etiquette norms linked to chivalry [15]. In fact, avoiding hitting hard at female players was a common practice in mixed doubles tennis in the 1960–1980s, while more recent research has shown that an important shift occurred in the early twenty-first century [15]. Although this study did not track the target of the final shot (i.e., whether male winners resulted from hitting at the opposing male or female cannot be determined), our findings show that when the penultimate shot is played by a female opponent, male players are more likely to produce a winning shot.

Second, sex differences in error production were context dependent. Females committed more forced errors overall (medium ES), replicating the defensive imbalance reported in mixed table-tennis [12]. However, this gap disappeared under favorable conditions. Specifically, once rallies from pairs who were ahead or ultimately winning the match were isolated, differences were not significant anymore, suggesting that a favorable condition or simply being part of a higher-skilled pair allows female athletes to defend as effectively as their male partners. From a coaching perspective, technical and tactical preparation should focus both on the coordination between male and female players, and on positioning strategies with related physical training. In particular, incorporating defensive simulations designed to improve pressure management, especially for female athletes, could be an effective way to strengthen both resilience and consistency in defense for all players.

Third, the sex of the opponent executing the penultimate stroke influenced rally outcomes. When the penultimate ball of the rally was played by an opposing female (PF condition), male players still produced extra winners and committed fewer forced errors (small to medium ES). In contrast, when the shot originated from a male opponent (PM condition), all sex differences vanished. One practical interpretation is that a female-hit approach may be driven at a lower pace, affording the male receiver better finishing opportunities, whereas male-generated hits neutralize that edge. Notably, this “penultimate-shot phenomenon” is mitigated among winning pairs, implying that better female strokes can erase this performance imbalance.

### 4.1. Practical Implications

The present findings seem to suggest a dual emphasis in training: (i) increasing females’ resistance to high-pressure exchanges through drills, for example, that rehearse rapid volley-block sequences under time constraints, and consequentially (ii) redistributing finishing responsibility, so that females are encouraged to close rallies, at least when the set-up ball comes from an opponent female. In addition, teams may benefit from scouting data: if the opposing pair tends to direct shots toward the female player after the opening rally exchanges, designing tactical patterns that allow the female partner to counter-attack could help neutralize the typical male-dominated winning shot.

### 4.2. Limitations and Future Research

The sample comprised only 17 elite matches, so generalizability to professional events, different playing styles (e.g., rally scoring vs. side-out), or the sub-elite and recreational population could be limited. All outcomes were coded manually from video without the chance to measure kinematic features (e.g., speed ball); adding ball-tracking metrics (speed, spin, landing zone) and time-motion variables would clarify why a male finishing advantage happens. Moreover, we cannot directly and separately determine the source of the female players’ better performance. While their outcomes tend to improve when the pair wins the match, it is unclear whether this reflects the female player’s individual skill or the male partner compensating for her weaknesses. Future work should also test whether the observed patterns persist when mixed pairs adopt set plays that deliberately invert traditional gender roles, or when final shots are executed from different court positions, for example, from the baseline or near the no-volley zone.

## 5. Conclusions

This study is the first to quantitatively examine technical, tactical, and time-motion characteristics in elite mixed doubles pickleball, in general and with a focus on sex-based dynamics, shot efficacy, and contextual influences. The present study has been able to demonstrate how mixed doubles pickleball currently mirrors historic trends in other racket sports: male players are the principal finishers, while females bear a larger share of forced errors under neutral or losing circumstances. However, these disparities tend to reduce or disappear when the pair is strong enough to lead or win, suggesting the technical and tactical, rather than intrinsic, nature of the gap. Taken together, the present findings suggest that mixed doubles pickleball may be partially shaped by sex-imbalanced game dynamics, potentially limiting the full effectiveness of the technical and tactical performance as well as the gender inclusivity in competitive play. From a practical standpoint, the findings of this study suggest that by coaching toward balanced finishing roles and pressure-tolerant defense, practitioners can move the discipline closer to true gender-equitable play and gain technical and tactical advantages, i.e., perform better and win more.

## Figures and Tables

**Table 1:** Descriptive values of the parameters considered for the time-motion analyses.

KPI	Mean ± Standard Deviation
Total match duration (min)	36.1 ± 12.3
Effective playing time (min)	17.5 ± 6.6
Work-to-rest ratio (WRR)	1:1.4 ± 0.2
Average rally duration (sec)	10.6 ± 1.8
Average recovery between rallies (sec)	11.4 ± 0.9

**Table 2:** Descriptive values (%; means ± dev.st.) between male and female final shot outcomes.

Overall						
	Overall		Penultimate Female		Penultimate Male	
	Male	Female	Male	Female	Male	Female
Forced errors	50.2 ± 10.1	61.2 ± 9.5	17.8 ± 7.9	22.4 ± 7.7	32.3 ± 7.7	38.7 ± 9.5
Unforced errors	21.5 ± 10.3	20.7 ± 9.3	9.4 ± 5.4	9.4 ± 5.7	10.7 ± 7.5	8.7 ± 6.4
Winners	28.3 ± 7.9	18.1 ± 6.7	15.7 ± 7.1	8.2 ± 5.1	12.6 ± 6.4	9.9 ± 3.8
**Winning the match (MoV)**						
Forced errors	47.6 ± 13.3	54.2 ± 11.0	18.0 ± 11.4	22.6 ± 11.2	29.6 ± 10.2	31.5 ± 13.6
Unforced errors	19.7 ± 10.5	20.5 ± 10.2	9.2 ± 5.6	9.8 ± 7.0	9.3 ± 7.9	8.0 ± 8.1
Winners	32.7 ± 12.9	12.2 ± 10.8	17.3 ± 7.7	12.2 ± 10.8	15.4 ± 10.2	13.0 ± 5.7
**Leading in the running score (MoA)**						
Forced errors	51.6 ± 12.5	56.9 ± 11.0	21.0 ± 10.8	24.1 ± 11.7	30.6 ± 11.1	32.2 ± 15.0
Unforced errors	18.6 ± 11.5	21.1 ± 8.5	9.4 ± 6.5	10.4 ± 6.8	7.7 ± 8.0	7.9 ± 8.7
Winners	29.8 ± 12.2	22.0 ± 9.4	16.4 ± 8.4	10.4 ± 7.4	16.4 ± 8.4	11.6 ± 6.2

## Data Availability

The datasets generated and analyzed during the current study are available in the Zenodo repository at https://doi.org/10.5281/zenodo.17086500.

## Supplementary Materials

The following supporting information can be downloaded at: https://www.mdpi.com/article/10.3390/sports13110397/s1, Table S1: Definition and description of the technical and tactical and time-motion KPIs.; Table S2: Paired-sample *t*-test results comparing male and female outcomes for forced errors (MFE vs. FFE), unforced errors (MUE vs. FUE), and winners (MW vs. FW), both overall (regardless of the penultimate shot) and separately based on whether the penultimate shot was played by a male (PM) or a female (PF). Table S3: Paired-sample *t*-test results comparing male and female outcomes for forced errors (MFE vs. FFE), unforced errors (MUE vs. FUE), and winners (MW vs. FW), both overall (regardless of the penultimate shot) and separately based on whether the penultimate shot was played by a male (PM) or a female (PF), considering only the shots of the pair that won the match. Table S4: Paired-sample *t*-test results comparing male and female outcomes for forced errors (MFE vs. FFE), unforced errors (MUE vs. FUE), and winners (MW vs. FW), both overall (regardless of the penultimate shot) and separately based on whether the penultimate shot was played by a male (PM) or a female (PF), considering only the shots made by the pair leading in the running score.
