# Supplementary material for: Is She or He the Key Player in Pickleball Mixed Doubles? A Pilot Study on Sex-Based Performance Profiles
Source: Sports (Basel). 2025 Nov 6;13(11):397. doi: 10.3390/sports13110397 (PMC12656059; doi:10.3390/sports13110397)
Supplement: Supplementary file 1 [file sports-13-00397-s001.zip › sports-3908246-supplementary.pdf]

Supplementary Table S1

S1.Definition and description of the technical and tactical and time-motion KPIs.

KPI	Description
O-MFE	Male forced error on the final shot by the male player, caused by effective opponent pressure rather than the player's own lapse, regardless of score margin, current advantage, or the penultimate shot.
O-FFE	Female forced error on the final shot by the female player, produced by effective opponent pressure, independent of score margin, current advantage, or the penultimate shot.
O-MUE	Male unforced error on the final shot by the male player in a favourable or neutral situation without notable opponent pressure, regardless of score margin, current advantage, or the penultimate shot.
O-FUE	Female unforced error on the final shot by the female player in a favourable or neutral situation without notable opponent pressure.
O-MW	Male winner: a decisive shot by the male player that ends the rally without an opponent error, irrespective of score margin, current advantage, or the penultimate shot.
O-FW	Female winner: a decisive shot by the female player that ends the rally without an opponent error.
PF-MFE	Male forced error under pressure when the penultimate shot was hit by the opposing female.
PF-FFE	Female forced error under pressure when the penultimate shot was hit by the opposing female.
PF-MUE	Male unforced error in a favourable/neutral context when the penultimate shot was hit by the opposing female.
PF-FUE	Female unforced error in a favourable/neutral context when the penultimate shot was hit by the opposing female.
PF-MW	Male winner when the penultimate shot was hit by the opposing female.
PF-FW	Female winner when the penultimate shot was hit by the opposing female.
PM-MFE	Male forced error under pressure when the penultimate shot was hit by the opposing male.
PM-FFE	Female forced error under pressure when the penultimate shot was hit by the opposing male.
PM-MUE	Male unforced error when the penultimate shot was hit by the opposing male.
PM-FUE	Female unforced error when the penultimate shot was hit by the opposing male.
PM-MW	Male winner when the penultimate shot was hit by the opposing male.
PM-FW	Female winner when the penultimate shot was hit by the opposing male.
W-O-MFE	Male forced error for the match-winning pair, same definition as O-MFE.
W-O-FFE	Female forced error for the match-winning pair, same definition as O-FFE.
W-O-MUE	Male unforced error for the match-winning pair, same definition as O-MUE.
W-O-FUE	Female unforced error for the match-winning pair, same definition as O-FUE.
W-O-MW	Male winner for the match-winning pair, same definition as O-MW.
W-O-FW	Female winner for the match-winning pair, same definition as O-FW.
W-PF-MFE	Male forced error for the match-winning pair when the penultimate shot was hit by the opposing female.
W-PF-FFE	Female forced error for the match-winning pair when the penultimate shot was hit by the opposing female.
W-PF-MUE	Male unforced error for the match-winning pair when the penultimate shot was hit by the opposing female.

W-PF-FUE	Female unforced error for the match-winning pair when the penultimate shot was hit by the opposing female.
W-PF-MW	Male winner for the match-winning pair when the penultimate shot was hit by the opposing female.
W-PF-FW	Female winner for the match-winning pair when the penultimate shot was hit by the opposing female.
W-PM-MFE	Male forced error for the match-winning pair when the penultimate shot was hit by the opposing male.
W-PM-FFE	Female forced error for the match-winning pair when the penultimate shot was hit by the opposing male.
W-PM-MUE	Male unforced error for the match-winning pair when the penultimate shot was hit by the opposing male.
W-PM-FUE	Female unforced error for the match-winning pair when the penultimate shot was hit by the opposing male.
W-PM-MW	Male winner for the match-winning pair when the penultimate shot was hit by the opposing male.
W-PM-FW	Female winner for the match-winning pair when the penultimate shot was hit by the opposing male.
AD-O-MFE	Male forced error for the pair currently leading the score, same definition as O-MFE.
AD-O-FFE	Female forced error for the leading pair, same definition as O-FFE.
AD-O-MUE	Male unforced error for the leading pair, same as O-MUE.
AD-O-FUE	Female unforced error for the leading pair, same as O-FUE.
AD-O-MW	Male winner for the leading pair, same as O-MW.
AD-O-FW	Female winner for the leading pair, same as O-FW.
AD-PF-MFE	Male forced error for the leading pair when the penultimate shot was hit by the opposing female.
AD-PF-FFE	Female forced error for the leading pair when the penultimate shot was hit by the opposing female.
AD-PF-MUE	Male unforced error for the leading pair when the penultimate shot was hit by the opposing female.
AD-PF-FUE	Female unforced error for the leading pair when the penultimate shot was hit by the opposing female.
AD-PF-MW	Male winner for the leading pair when the penultimate shot was hit by the opposing female.
AD-PF-FW	Female winner for the leading pair when the penultimate shot was hit by the opposing female.
AD-PM-MFE	Male forced error for the leading pair when the penultimate shot was hit by the opposing male.
AD-PM-FFE	Female forced error for the leading pair when the penultimate shot was hit by the opposing male.
AD-PM-MUE	Male unforced error for the leading pair when the penultimate shot was hit by the opposing male.
AD-PM-FUE	Female unforced error for the leading pair when the penultimate shot was hit by the opposing male.
AD-PM-MW	Male winner for the leading pair when the penultimate shot was hit by the opposing male.
AD-PM-FW	Female winner for the leading pair when the penultimate shot was hit by the opposing male.
Total playing time (min)	Effective playing time + total recovery time
Effective playing time (min)	Effective playing time from the serve to the point scored
Work-to-rest ratio (WRR)	Average of the ratios between rally duration and the following recovery time
Average rally duration (sec)	Average duration of the time intervals from the serve to the point(s) scored
Average recovery between rallies (sec)	Average duration of the time intervals from the point scored to the next serve(s)

Supplementary Table S2

S2. Paired-sample t-test results comparing male and female outcomes for forced errors (MFE vs FFE), unforced errors (MUE vs FUE), and winners (MW vs FW), both overall (regardless of the penultimate shot) and separately based on whether the penultimate shot was played by a male (PM) or a female (PF).

		**statistic**	**df**	**p**	**Mean difference**	**SE difference**	**Effect Size**
O-MFE	O-FFE	-3.4121	16.0	0.004	-0.11118	0.0326	-0.8275
O-MUE	O-FUE	0.2413	16.0	0.812	0.00765	0.0317	0.0585
O-MW	O-FW	4.1049	16.0	< .001	0.10235	0.0249	0.9956
PF-MFE	PF-FFE	-2.2027	16.0	0.043	-0.04706	0.0214	-0.5342
PF-MUE	PF-FUE	-0.0765	16.0	0.940	-0.00118	0.0154	-0.0185
PF-MW	PF-FW	3.3138	16.0	0.004	0.07529	0.0227	0.8037
PM-MFE	PM-FFE	-1.9643	16.0	0.067	-0.06412	0.0326	-0.4764
PM-MUE	PM-FUE	0.7780	16.0	0.448	0.01824	0.0234	0.1887
PM-MW	PM-FW	1.5265	16.0	0.146	0.02882	0.0189	0.3702

Supplementary Table S3

S3. Paired-sample t-test results comparing male and female outcomes for forced errors (MFE vs FFE), unforced errors (MUE vs FUE), and winners (MW vs FW), both overall (regardless of the penultimate shot) and separately based on whether the penultimate shot was played by a male (PM) or a female (PF), considering only the shots of the pair that won the match.

		**statistic**	**df**	**p**	**Mean difference**	**SE difference**	**Effect Size**
W-O-MFE	W-O-FFE	-1.618	16.0	0.125	-0.06529	0.0403	-0.3925
W-O-MUE	W-O-FUE	-0.256	16.0	0.801	-0.00941	0.0368	-0.0621
W-O-MW	W-O-FW	4.928	16.0	< .001	0.20471	0.0415	1.1951
W-PF-MFE	W-PF-FFE	-1.279	16.0	0.219	-0.04471	0.0350	-0.3102
W-PF-MUE	W-PF-FUE	-0.375	16.0	0.713	-0.00765	0.0204	-0.0909
W-PF-MW	W-PF-FW	1.369	16.0	0.190	0.05000	0.0365	0.3321
W-PM-MFE	W-PM-FFE	-0.475	16.0	0.641	-0.02059	0.0434	-0.1152
W-PM-MUE	W-PM-FUE	0.601	16.0	0.556	0.01235	0.0206	0.1458
W-PM-MW	W-PM-FW	0.938	16.0	0.362	0.02353	0.0251	0.2276

Supplementary Table S4

S4. Paired-sample t-test results comparing male and female outcomes for forced errors (MFE vs FFE), unforced errors (MUE vs FUE), and winners (MW vs FW), both overall (regardless of the penultimate shot) and separately based on whether the penultimate shot was played by a male (PM) or a female (PF), considering only the shots made by the pair leading in the running score.

		statistic	df	p	Mean difference	SE difference	Effect Size
AD-O-MFE	AD-O-FFE	-1.2275	16.0	0.237	-0.05294	0.0431	-0.2977
AD-O-MUE	AD-O-FUE	-0.7313	16.0	0.475	-0.02529	0.0346	-0.1774
AD-O-MW	AD-O-FW	2.2981	16.0	0.035	0.07824	0.0340	0.5574
AD-PF-MFE	AD-PF-FFE	-0.9299	16.0	0.366	-0.02941	0.0316	-0.2255
AD-PF-MUE	AD-PF-FUE	-0.5436	16.0	0.594	-0.01118	0.0206	-0.1318
AD-PF-MW	AD-PF-FW	2.1387	16.0	0.048	0.06059	0.0283	0.5187
AD-PM-MFE	AD-PM-FFE	-0.3581	16.0	0.725	-0.01647	0.0460	-0.0869
AD-PM-MUE	AD-PM-FUE	-0.0604	16.0	0.953	-0.00176	0.0292	-0.0147
AD-PM-MW	AD-PM-FW	1.8907	16.0	0.077	0.04882	0.0258	0.4586
